# Diseases of the Liver, Gall-Bladder, and Biliary System

**Published:** 1897-12

**Authors:** 


**Diseases of the Liver, Gall-Bladder, and Biliary System.** By H. J. Waring, M.S. Pp. xiv., 385. Edinburgh: Young J. Pentland. 1897.

The essay on which this book is founded was awarded the Jacksonian prize of the Royal College of Surgeons. Mr. Waring has collected information bearing on his subject from all sources, and has clearly formulated methods of operation advisable in the various diseases with which surgery can deal, and he has given us the latest views as to pathology, and a carefully prepared description of symptoms; but the work lacks advice as to diagnosis and treatment based on the writer's own experience. Mr. Waring has performed experimental operations in the laboratories of the College of Surgeons; but valuable as these are they will not compensate for information based on the experience gained by treating these diseases, or for the lack of illustrative cases from the author's practice.

The book opens with an account of the anatomy and physiology of the liver; and the relations of the bile-duct in the gastro-hepatic omentum—of such great practical importance to the surgeon—are clearly described and figured. The arrangement of mucous membrane at the commencement of the cystic duct is well shown in a diagram (after Testut), and it is enough to look at the diagram to see how easily a bougie used to explore the ducts may be caught by the so-called "spiral valve." Operations are described in detail, separately, at the end of the book. No one can read this work without being struck by the way in which surgery now deals with those diseases of the liver and gall-tracts, which until late years remained within the domain of the physician. As was truly said not long ago, the surgeon of the present day has very largely become an operating physician.

We must now pass on to notice more in detail some of the contents of the book. The chapter on hepatectomy, or the removal of growths from the liver, is interesting and instructive, as it gives us a description of recent attempts in this direction, and the result of the author's own experiments. Information is also given about movable liver—a condition only lately recognised—and its fixation by operation is described. In the account of cholecystotomy we fail to find any directions for dealing with cases in which there is great difficulty in bringing the gall-bladder to the anterior abdominal wall; and in discussing the desirability of performing "ideal cholecystotomy," we do not think enough importance is made of the patency of the gall-ducts. Suturing the incision in the gall-bladder and leaving a drainage tube in the wound in the abdominal wall passed down to the sutured gall-bladder, is not what is usually understood as "ideal cholecystotomy." In "ideal cholecystotomy" both the incision in the gall-bladder and in the abdominal wall are closed. In cholecystenterostomy we are glad to see the author speaks so highly of the use of Murphy's button, and we feel sure that his recommendation to put a row of sutures around the junction made by the button is a wise one, though not advocated by Murphy himself. We fail to find any mention of the risk of kinking of the gut in this operation, which has led to fatal obstruction in at least one recorded case. Mr. Waring considers this operation indicated when the common duct is blocked by malignant growth, but he admits that the operation when performed for such a condition is a very fatal one. We are glad to see that he speaks with hesitation about the advantage of crushing gall-stones in the ducts, and lays stress on the dangers of the procedure. The operation which the author describes for hepatic abscess seems to us rather unsafe. Instead of carefully stitching the liver to the parietal peritoneum before opening the abscess, he advises that the liver should be packed round with sponges before the abscess is opened, and then that the exposed surface of the liver and adjacent portion of the peritoneum should be thoroughly irrigated. If the sponges are packed round so as to prevent the entrance of pus into the peritoneal cavity, irrigation will be worse than useless; and if they are not, nothing short of irrigation of the whole peritoneal cavity will be called for. In discussing the diagnosis of rupture of the liver, the difficulties which are met with in actual practice seem hardly to be sufficiently described—for instance, the differential diagnosis from ruptured intestine, and the difficulty of distinguishing the symptoms of internal hemorrhage from those of shock, are not considered.

We have read this book with great interest. Even now it is a valuable contribution to the ever advancing knowledge which during the last ten years has placed abdominal surgery in so prominent and satisfactory a position; but its usefulness will be immensely increased when, in later editions, the author is able to add to it the record of his own experience.

